# Co-Expression of TWIST1 and ZEB2 in Oral Squamous Cell Carcinoma Is Associated with Poor Survival

**DOI:** 10.1371/journal.pone.0134045

**Published:** 2015-07-27

**Authors:** Yink Heay Kong, Sharifah Nurain Syed Zanaruddin, Shin Hin Lau, Anand Ramanathan, Thomas George Kallarakkal, Vui King Vincent-Chong, Wan Mahadzir Wan Mustafa, Mannil Thomas Abraham, Zainal Ariff Abdul Rahman, Rosnah Binti Zain, Sok Ching Cheong

**Affiliations:** 1 Department of Oro-maxillofacial Surgery and Medical Sciences, Faculty of Dentistry, University of Malaya, Kuala Lumpur, Malaysia; 2 Oral Cancer Research Team, Cancer Research Initiatives Foundation, Subang Jaya, Selangor, Malaysia; 3 Stomatology Unit, Institute for Medical Research, Kuala Lumpur, Malaysia; 4 Oral Cancer Research & Coordinating Centre, University of Malaya, Kuala Lumpur, Malaysia; 5 Department of Oral & Maxillofacial Surgery, Hospital Kuala Lumpur, Kuala Lumpur, Malaysia; 6 Department of Oral & Maxillofacial Surgery, Tengku Ampuan Rahimah Hospital, Klang, Malaysia; University of North Carolina School of Medicine, UNITED STATES

## Abstract

Oral squamous cell carcinoma (OSCC) is an aggressive disease accounting for more than 260,000 cancer cases diagnosed and 128,000 deaths worldwide. A large majority of cancer deaths result from cancers that have metastasized beyond the primary tumor. The relationship between genetic changes and clinical outcome can reflect the biological events that promote cancer’s aggressive behavior, and these can serve as molecular markers for improved patient management and survival. To this end, epithelial-mesenchymal transition (EMT) is a major process that promotes tumor invasion and metastasis, making EMT-related proteins attractive diagnostic biomarkers and therapeutic targets. In this study, we used immunohistochemistry to study the expression of a panel of transcription factors (TWIST1, SNAI1/2, ZEB1 and ZEB2) and other genes intimately related to EMT (CDH1 and LAMC2) at the invasive tumor front of OSCC tissues. The association between the expression of these proteins and clinico-pathological parameters were examined with Pearson Chi-square and correlation with survival was analyzed using Kaplan Meier analysis. Our results demonstrate that there was a significant differential expression of CDH1, LAMC2, SNAI1/2 and TWIST1 between OSCC and normal oral mucosa (NOM). Specifically, CDH1 loss was significantly associated with Broder’s grading, while diffused LAMC2 was similarly associated with non-cohesive pattern of invasion. Notably, co-expression of TWIST1 and ZEB2 in OSCC was significantly associated with poorer overall survival, particularly in patients without detectable lymph node metastasis. This study demonstrates that EMT-related proteins are differentially expressed in OSCC and that the co-expression of TWIST1 and ZEB2 could be of clinical value in identifying patients with poor survival for appropriate patient management.

## Introduction

Despite commendable advancements in the prevention, detection and treatment of cancer, oral squamous cell carcinoma (OSCC) still remains a significant global health burden, accounting for over 260,000 diagnosed cases and 128,000 deaths worldwide [1]. Cancer metastasis which arises when cancer cells spread from the primary tumor to form new tumors in distant organs is the primary cause of death in most cancer patients. This process involves changes in tumor tissue architecture that promote invasion and metastasis and is regulated through a mechanism known as epithelial-mesenchymal transition (EMT) [2]. The activation of EMT facilitates cancer epithelial cells to undergo dedifferentiation to a mesenchymal phenotype and in the process gain a metastatic capability that allows these cells to invade through the basement membrane and migrate to regional lymph node or distant organs [2]. The emerging roles of some key EMT-related proteins in cancer progression and their close correlation with clinico-pathological parameters make them attractive targets for developing diagnostic biomarkers and therapies [3]. However, with the complexity of signaling networks involved in EMT, an in depth understanding of which of the related proteins are important in this process would hold value in translating this information for clinical benefit.

While the initiation of EMT is a complex process involving many complex pathways [2], the loss of cell adhesion among epithelial cells is a key event, defined by the down-regulation of E-cadherin (CDH1). Expression of CDH1 is regulated by transcription factors including SNAI1, SNAI2, ZEB1, ZEB2 and TWIST1 which act either directly or indirectly on the CDH1 promoter [2, 4]. These transcription factors form signaling networks that could initiate and sustain the mesenchymal phenotypes of tumor cells [3], and therefore the expression of these proteins could define EMT occurrence in a tumor setting. Most notably, LAMC2, the γ2 chain of Laminin 332, has been shown to be involved in tumor migration and invasion processes [5, 6]. Furthermore, overexpression of LAMC2 in OSCC is also associated with loss of cell polarity [7], likely suggesting a role in tumor spread.

EMT occurrence demonstrated by the expression of EMT-associated proteins as described above has been reported for many different cancer types including head and neck cancer [3, 4, 8]. Some of these studies broadly focused on interrogating the invasive front of the tumor, an area where tumor cells have been reported to have the highest malignant potential [9–11]. In these studies, CDH1 loss and overexpression of SNAI1, TWIST1 and LAMC2 were observed at the invasive front of the tumors, particularly in single or cords of tumor cells detaching from the tumor mass [7, 12–23]. Additionally, loss of CDH1, and overexpression of SNAI1 and LAMC2 at the invasive front have been associated with metastasis and worse prognosis in oral, esophageal and lung cancer [15, 19–21]. While the expression of these EMT drivers have been well studied in many cancers, in OSCC this process has largely been defined by CDH1 switching (from E-cadherin to N-cadherin), and information regarding expression patterns of other EMT markers including the EMT transcription factors in OSCC is limited. Furthermore, in light of regional metastasis being the single most important prognostic factor for OSCC [24], there is a need to determine the nature of the transcription factors that may be involved in cancer progression and how they may impact clinical outcome.

In this study, using immunohistochemistry, we sought to understand the expression pattern of EMT-related markers (CDH1, LAMC2, SNAI1/2, TWIST1, ZEB1 and ZEB2) at the invasive front of OSCC archival specimens. From our observation, we demonstrated a significant difference in the expression of several key proteins (CDH1, LAMC2, SNAI1/2 and TWIST1) between OSCC and non-malignant oral mucosa (NOM) tissues. Specifically, CDH1 loss at the invasive front was associated with Broder’s grading and diffused LAMC2 was associated with non-cohesive pattern of invasion. Notably, co-expression of transcription factors, TWIST1 and ZEB2, was associated with a poorer patient overall survival, particularly in cohorts without detectable lymph node metastasis, likely suggesting a clinical utility in identifying patients with poor survival for appropriate patient management.

## Materials and Methods

### Archival OSCC tumor tissues and construction of tissue microarray (TMA)

In this retrospective study, 148 formalin-fixed paraffin-embedded (FFPE) tissues blocks including histopathologically confirmed OSCC (n = 87) and NOM tissues were included (n = 61; consisting of denture hyperplasia, impacted third molar gingival tissue and matching normal mucosa tissue adjacent to the tumor). OSCC tissues included in this study were diagnosed between 2003 and 2011 at University of Malaya and the Institute for Medical Research (IMR) and followed-up up till July 2013. Primary surgical lesions with identifiable invasive tumor front were included, and clinical information (*i*.*e*. tumor stage, tumor size, lymph node metastasis, pattern of invasion (POI), Broder’s grading and overall survival) from these patients were obtained from the Malaysian Oral Cancer Database & Tissue Bank System (MOCDTBS) at the Oral Cancer Research and Coordinating Centre (OCRCC), University of Malaya, Malaysia [25], and the IMR (Table 1). Tissues from metastatic and recurrent lesions were excluded from this study. Written informed consent was obtained from the participants for their clinical records to be used in this study and all patient information was anonymized and de-identified prior to analysis. This study was approved by the Institutional Review Board, of the Faculty of Dentistry, University of Malaya (Ethical Clearance number: DF OP1109/0084(L)).

**Table 1:** Demographics and clinico-pathological characteristics of patients.

Tissue type		Total samples (n = 148)	(%)
**Non-malignant**	** **	61	41.2
	Denture hyperplasia	7	11.5
	Impacted third molar gingival	20	32.8
	Adjacent normal	34	55.7
**OSCC**		87	58.8
** **	** **	** **	** **
**Variables**		**OSCC (n = 87)**	**(%)**
**Gender**	Male	34	39.1
** **	Female	53	60.9
**Age**	Range	28 to 86	
** **	Mean	59.1	
**Tumor Site**	Cheek	33	37.9
** **	Tongue	33	37.9
** **	Gum	9	10.3
** **	Others (lip and palate)	12	13.8
**Tumor Size**	Tis	1	1.1
** **	T1 & T2	39	44.8
** **	T3 & T4	47	54.0
**Node Stage**	Nx	2	2.3
** **	N0	47	54.0
** **	N1, N2, N3	38	43.7
**pTNM Stage**	Carcinoma *in situ*	1	1.1
	I & II (early)	28	32.2
	III & IV (late)	58	66.7
**Pattern of**	Cohesive	21	24.1
**Invasion**	Non-cohesive	66	75.9
**Broders' Grading**	Well differentiated	40	46.0
	Moderately/Poorly differentiated	42	48.3
	N/A	5	5.7
**Treatment**	Surgery alone	18	20.7
** **	Surgery plus chemotherapy and/or radiotherapy	44	50.6
** **	N/A	25	28.7
**Overall survival**	Range (months)	1 to 90	
** **	Median	20	

Abbreviations: OSCC, oral squamous cell carcinoma; n, number of specimens; N/A, not available.

Three representative areas of invasive tumor fronts in OSCC specimens and two representative areas of the NOM tissues were identified by board certified pathologists (AR, TGK, RBZ and LSH) for the construction of the tissue microarray (TMA). Tissue cores of 1.0 mm diameter were extracted from the identified invasive tumor front areas using MiniCore 2 and TMA designer 2 (Alphelys, France). Each core was then re-embedded into a recipient paraffin block in an asymmetric grid-like arrangement [26]. The TMAs were then sectioned at 4 μm for hematoxylin and eosin (H&E) or immunohistochemical (IHC) staining.

### Selection of candidate EMT-related markers and immunohistochemical staining

The occurrence of EMT has been associated with the loss of CDH1, and overexpression of proteins including LAMC2 and EMT transcription factors SNAI1, SNAI2, TWIST1, ZEB1 and ZEB2 [3, 4, 7, 22, 27, 28]. The expressions of these proteins were examined by immunohistochemical staining (IHC).

IHC staining was performed according to manufacturer’s protocols for DakoCytomation REAL EnVision Detection System-HRP or the Universal Biotinylated Link LSAB System-HRP (Dako, Denmark) as previously described [29]. Information on the use of antibodies against the EMT-related markers studied (SNAI1/2, TWIST1, ZEB1, and ZEB2) is summarized in S1 Table. FFPE tissues from breast, colorectal, cervical and ovarian cancers were used as positive controls in these experiments as suggested on the data sheet for each of the antibodies used (S1A–S1H Fig). Ten random whole tissue sections were also immuno-stained against these EMT-related markers to confirm that similar staining patterns and intensities were observed in these whole tissue sections and their respective TMA cores.

### Evaluation of immunohistochemical staining

Each core was considered to be suitable for evaluation if it contained epithelial cells (tumor and normal). Only the immunohistochemical staining of the epithelial cells were graded, and for those samples with no epithelial cells in all of the corresponding three tissue cores, evaluation was not performed. The evaluation of the immunohistochemical staining was carried out by three pathologists (AR, TGK and LSH), who were essentially blind to all histopathological and clinical data and any discrepancies were discussed to reach a consensus agreement to a definitive score.

As CDH1 expression was observed in both membrane and cytoplasm of epithelial cells, only membranous staining was evaluated, as only the loss CDH1 expression at this location is indicative of EMT occurrence [30] and is predominantly evaluated to determine the prognostic significance of CDH1 [12, 21, 31, 32]. CDH1 staining was also categorized into uniformly positive, uniformly negative and heterogeneous as previously reported [33]. Uniformly positive CDH1 expression refers to expression of membranous CDH1 in all layers of the epithelium while heterogeneous CDH1 expression indicates gradual loss of membranous CDH1 in tumor cells [33]. Also, cytoplasmic LAMC2 staining was categorized into diffused (cytoplasmic LAMC2 staining in all layers of cells), peripheral (LAMC2 staining at peripheral layer but not at the center of the tumor island) and negative (no cytoplasmic expression and/or expression at the basement membrane lining the normal epithelium) as reported by others [7]. The intensity of nuclear expression was evaluated for EMT transcription factors, where 0 = negative staining, 1 = weak staining, 2 = moderate staining, 3 = strong staining [34–36]. The extent of positive staining was not taken into account for the overall analyses largely due to the percentage derived from TMA tissue cores would only represent a small proportion of the invasive front area and not the whole tissue section. Overall, the scores were consistent in all the cores from each specimen. However, where there were differences among the three tissue cores, the highest intensity or worse pattern of staining (*e*.*g*. heterogeneous CDH1 and diffused LAMC2 staining) was taken. The evaluation scores of EMT transcription factors were further separated into binary scores. For SNAI1/2, this separation was based on median intensity as described by Galvan *et al*. [34]. The median of intensity score for SNAI1/2 in this study was 3, hence SNAI1/2 scores were divided into 0, 1, 2 versus 3. Similarly, the median of intensity score for ZEB2 and TWIST1 was computed to be 0, hence the scores were divided into negative (0) versus positive (1, 2 and 3) as described by others [35, 36]. None of the samples expressed ZEB1, therefore, binary score of ZEB1 could not be computed and statistical analyses could not be conducted for ZEB1. The evaluation scores for CDH1 and LAMC2 were also further separated into binary scores. CDH1 scores were divided into preserved (uniformly positive) and reduced (uniformly negative/heterogeneous) while LAMC2 scores were divided into diffused and non-diffused (negative/basement membrane/peripheral). The criteria for evaluation of all markers are summarized in S1 Table.

### Statistical analysis

Following the “Reporting Recommendations for Tumor Marker Prognostic Studies” (REMARK) criteria closely, the minimum number of samples that are needed for this study was determined using a sample size calculator computing for a two-sided chi-square analysis. Using data from published studies, we demonstrated that the number of specimens included in this study was adequate, as the minimum required number of normal and OSC tissues were 56 and 10 respectively, to achieve a statistical power of 80% and significant *p* value of 0.05 (S2 Table). Statistical analyses were performed using SPSS for Windows 16.0 (IBM Inc., New York, USA). Pearson Chi-square test was performed to determine the statistical associations of the expression of EMT-related markers with different tissue types (*i*.*e*. NOM and OSCC) and clinico-pathological parameters (*i*.*e*. tumor stage, tumor size, node metastasis, pattern of invasion (POI) and Broders’ grading). Kaplan-Meier survival analysis was used to correlate survival rates with EMT-related proteins’ expressions, and the survival probability differences were compared by the log-rank test. Cox regression multivariate analyses were also performed to adjust for other factors that could influence the patient’s survival including tumor stage, pattern of invasion and lymph node metastasis. Any missing values were omitted from the analysis and a *p*-value <0.05 was considered to be statistically significant.

## Results

### Demographics and clinico-pathological characteristics of OSCC patients

A total of 148 tissue samples including 87 OSCC and 61 NOM tissues were analyzed. The demographics and clinico-pathological information on the OSCC patients are included in Table 1. The follow-up survival data were available for 79/87 patients (90.8%) with the maximum follow-up period of 90 months and a mean and median survival of 26.7 and 20 months respectively (Table 1).

### EMT-related markers were differentially expressed in OSCC and NOM tissues

**Fig 1:**
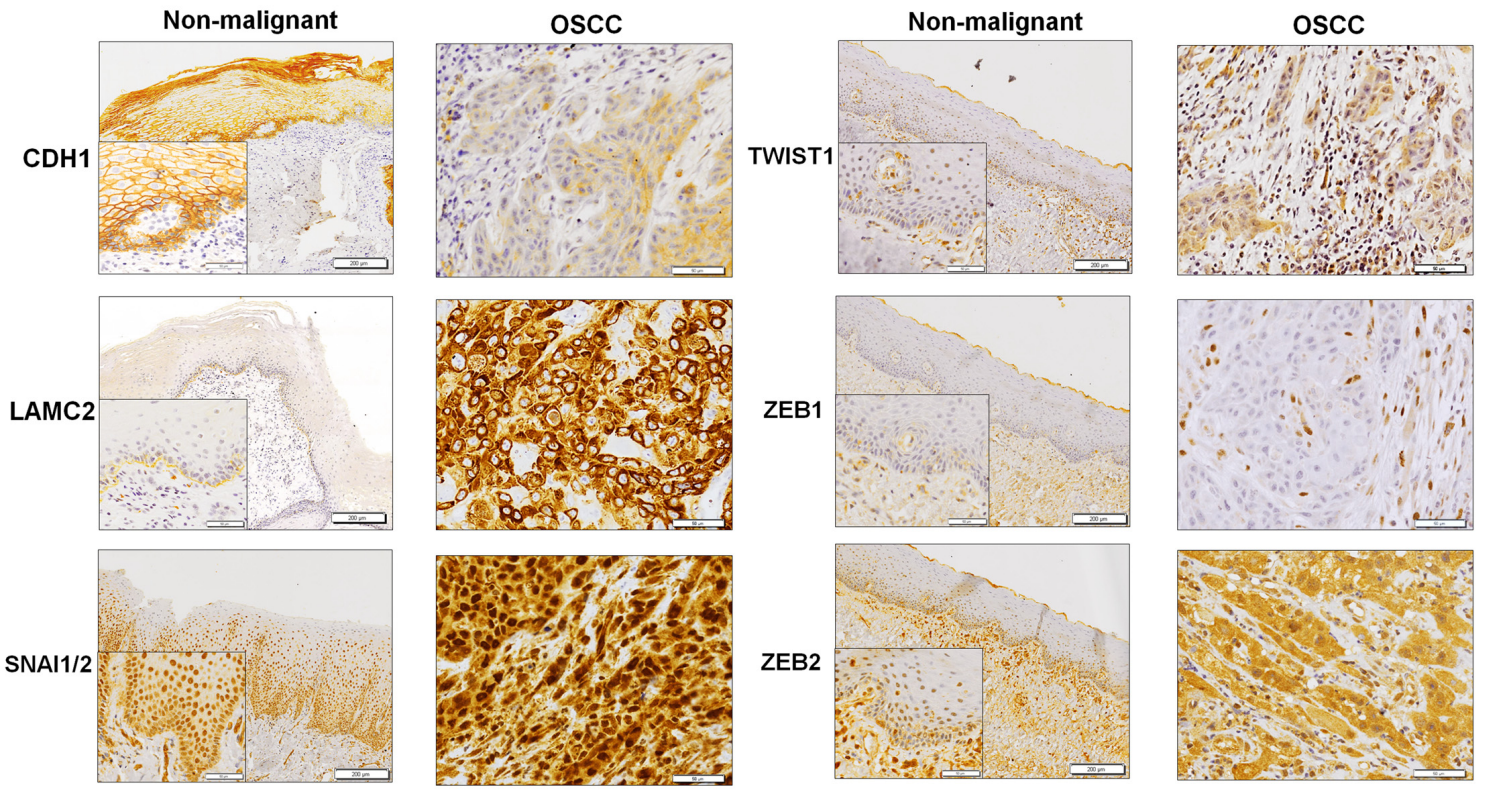
Representative immunostaining of EMT-related markers in non-malignant oral mucosa (Original magnification: 40X, 200X-Inset) and oral squamous cell carcinoma tissues (OSCC; Original magnification: 200X).

Before investigating the expression of EMT-related markers in OSCC and NOM tissues, we first sought to demonstrate that the staining pattern and intensity was broadly similar between ten random whole tissue sections and their respective TMA cores extracted from these sections (S2 Fig). On the TMA, an average of 129 (87.2%) tissue samples was deemed as interpretable for analyses. Among all the 6 markers tested, the expression of CDH1, SNAI1/2, LAMC2 and TWIST1, were noted to be differentially expressed between NOM and OSCC tissues (Fig 1; Table 2). Reduced CDH1 was observed in 64.9% of OSCC tissues compared to 20.7% of NOM tissues (*p* < 0.001; Table 2). The pattern of LAMC2 staining differed significantly in NOM and OSCC tissues (Fig 1) where 98% of NOM tissues had non-diffused staining and 52.4% of the OSCC had diffused staining (*p* < 0.001; Table 2).

**Table 2:** Differential expression of EMT-related proteins in non-malignant and OSCC tissues.

**Tissue Type**			**CDH1**	
	**n**	**Preserved (%)**	**Reduced (%)**	***p*-value**
**NOM**	58	46 (79.3)	12 (20.7)	
**OSCC**	77	27 (35.1)	50 (64.9)	<0.001*
**Tissue Type**	** **		**LAMC2**	
	**n**	**Non-diffused (%)**	**Diffused (%)**	***p*-value**
**NOM**	49	48 (98.0)	1 (2.0)	
**OSCC**	82	39 (47.6)	43 (52.4)	<0.001*
**Tissue Type**			**SNAI1/2**	
	** n**	**Weak (%)**	**Strong (%)**	***p*-value**
**NOM**	49	32 (65.3)	17 (34.7)	
**OSCC**	75	22 (29.3)	53 (70.7)	<0.001*
**Tissue Type**			**TWIST1**	
	** n**	**Negative (%)**	**Positive (%)**	***p*-value**
**NOM**	51	46 (90.6)	5 (9.4)	
**OSCC**	77	57 (74.0)	20 (26.0)	0.024*
**Tissue Type**			**ZEB2**	
	**n **	**Negative (%)**	**Positive (%)**	***p*-value**
**NOM**	53	31 (58.5)	22 (41.5)	
**OSCC**	77	35 (45.5)	42 (54.5)	0.144
**Tissue Type**			**ZEB1**	
	** n**	**Negative (%)**	**Positive (%)**	***p*-value**
**NOM**	51	51 (100.0)	0 (0.0)	
**OSCC**	73	73 (100.0)	0 (0.0)	N/A

Abbreviations: NOM, non-malignant oral mucosa; OSCC, oral squamous cell carcinoma; n, number of specimens.

Data were analyzed by Pearson's Chi-Square test.

*p < 0.05.

Expression of SNAI1/2, ZEB2 and TWIST1 were observed in the nucleus as well as the cytoplasm of epithelial cells (Fig 1), however, only nuclear staining in normal and tumor epithelial cells was evaluated as these proteins are expected to regulate transcription [37]. SNAI1/2 nuclear expression was noted to be with stronger intensity in a large majority of OSCC tissues (70.7%) compared to NOM tissues (34.7%; *p* < 0.001; Table 2). Despite the low prevalence, TWIST1 expression was observed to be ~3 fold elevated in OSCC compared to NOM tissues (*p* = 0.024; Table 2). Surprisingly, there was no significant difference in ZEB2 expression between NOM and OSCC tissues (*p* = 0.144; Table 2). ZEB1 expression was not observed in the nucleus of epithelial cells in NOM nor OSCC tissues although positive staining was observed in the inflammatory cells, fibroblast, muscle and endothelial cells in the stromal compartments surrounding the tumor cells (Fig 1). To ensure that the absence of ZEB1 staining was not due to quality of antibody used or experimental conditions, we thus stained for ZEB1 expression in breast cancer tissues that have been previously reported to express the protein [38]. Positive nuclear ZEB1 expression was observed in epithelial cells of breast cancer tissues (S1G Fig) confirming that NOM and OSCC inherently do not express this transcription factor.

### Association of EMT markers expression with clinico-pathological parameters

The association between the expressions of EMT-related markers with patients’ clinico-pathological parameters was also examined. As the loss of CDH1 is now considered as one of the hallmarks of EMT, we anticipated that reduced CDH1 expression would be observed in the majority of poorly differentiated tumors. Indeed, CDH1 expression was significantly associated with Broder’s grading where more than 80% of moderately/poorly differentiated tumors exhibited reduced CDH1 expression (*p* = 0.003, Table 3). As SNAI1/2, TWIST1 and ZEB2 are known to repress CDH1 expression, it is reasonable to expect to observe high expression levels of these factors being similarly associated with parameters that are associated with CDH1 loss. However, the expression of these transcription factors were not directly associated with the differentiation status of tumor cells and consistent with this observation, significant association between the expression of CDH1 and transcription factors was not demonstrated (S3 Table).

**Table 3:** Association between expression of EMT-related proteins with clinico-pathological parameters.

**Clinico-pathological parameters**				**CDH1**			
		**n**	**Preserved (%)**	**Reduced (%)**	***p*-value**	**Odds Ratio**	**95% confidence interval**
**Tumor Size**	Tis, T1,T2	35	11 (31.4)	24 (68.5)			
	T3,T4	42	16 (38.1)	26 (61.9)	0.542	1.343	0.521–3.462
**Node Metastasis**	Negative	42	15 (35.7)	27 (64.3)			
	Positive	33	10 (30.3)	23 (69.7)	0.622	0.783	0.295–2.074
**Tumor Stage**	Early	24	7 (29.2)	17 (70.8)			
	Late	53	20 (37.7)	33 (62.3)	0.465	1.472	0.520–1.167
**Pattern of Invasion**	Cohesive	19	10 (52.6)	9 (47.4)			
	Non-cohesive	58	17 (29.3)	41 (70.7)	0.064	0.213	0.074–0.612
**Broder's grading**	Well	35	18 (51.4)	17 (48.6)			
	Moderate/poor	38	7 (18.4)	31 (81.6)	0.003*	0.373	0.129–1.081
**Clinico-pathological parameters**				**LAMC2**			
		**n**	**Non-diffused (%)**	**Diffused (%)**	***p*-value**	**Odds Ratio**	**95% confidence interval**
**Tumor Size**	Tis, T1,T2	40	20 (50.0)	20 (50.0)			
	T3,T4	42	19 (45.2)	23 (54.8)	0.666	1.211	0.508–2.884
**Node Metastasis**	Negative	47	24 (51.1)	23 (48.9)			
	Positive	33	13 (39.4)	20 (60.6)	0.303	1.605	0.651–3.959
**Tumor Stage**	Early	29	15 (51.7)	14 (48.3)			
	Late	53	24 (45.3)	29 (54.7)	0.577	1.295	0.523–3.207
**Pattern of Invasion**	Cohesive	21	15 (71.4)	6 (28.6)			
	Non-cohesive	61	24 (39.3)	37 (60.7)	0.011*	3.854*	1.313–11.317*
**Broder's grading**	Well	38	20 (52.6)	18 (47.4)			
	Moderate/poor	39	14 (35.9)	25 (64.1)	0.139	1.984	0.796–4.944
**Clinico-pathological parameters**				**SNAI 1/2**			
		**n**	**Weak (%)**	**Strong (%)**	***p*-value**	**Odds Ratio**	**95% confidence interval**
**Tumor Size**	Tis, T1,T2	38	9 (23.7)	29 (76.3)			
	T3,T4	37	13 (35.1)	24 (64.9)	0.276	0.573	0.209–1.569
**Node Metastasis**	Negative	42	12 (28.6)	30 (71.4)			
	Positive	31	8 (25.8)	23 (74.2)	0.793	1.150	0.404–3.275
**Tumor Stage**	Early	27	9 (33.3)	18 (66.7)			
	Late	48	13 (27.1)	35 (72.9)	0.568	1.346	0.484–3.742
**Pattern of Invasion**	Cohesive	20	9 (45.0)	11 (55.0)			
	Non-cohesive	55	13 (23.6)	42 (76.4)	0.072	2.643	0.899–7.772
**Broder's grading**	Well	32	13 (40.6)	19 (59.4)			
	Moderate/poor	38	8 (21.1)	30 (78.9)	0.075	2.566	0.896–7.344
**Clinico-pathological parameters**				**TWIST1**			
		**n**	**Negative (%)**	**Positive (%)**	***p*-value**	**Odds Ratio**	**95% confidence interval**
**Tumor Size**	Tis, T1,T2	39	28 (71.8)	11 (28.2)			
	T3,T4	38	29 (76.3)	9 (23.7)	0.651	0.790	0.284–2.196
**Node Metastasis**	Negative	43	33 (76.7)	10 (23.3)			
	Positive	32	24 (75.0)	8 (25.0)	0.861	1.100	0.378–3.201
**Tumor Stage**	Early	28	20 (71.4)	8 (28.6)			
	Late	49	37 (75.5)	12 (24.5)	0.694	0.811	0.285–2.310
**Pattern of Invasion**	Cohesive	21	15 (71.4)	6 (28.6)			
	Non-cohesive	56	42 (75.0)	14 (25.0)	0.75	0.833	0.271–2.563
**Broder's grading**	Well	34	23 (67.6)	11 (32.4)			
	Moderate/poor	38	30 (78.9)	8 (21.1)	0.277	0.558	0.193–1.610
**Clinico-pathological parameters**				**ZEB2**			
		**n**	**Negative (%)**	**Positive (%)**	***p*-value**	**Odds Ratio**	**95% confidence interval**
**Tumor Size**	Tis, T1,T2	37	15 (40.5)	22 (59.5)			
	T3,T4	40	20 (50.0)	20 (50.0)	0.405	0.682	0.276–1.682
**Node Metastasis**	Negative	43	20 (46.5)	23 (53.5)			
	Positive	32	14 (43.8)	18 (56.3)	0.812	1.118	0.445–2.806
**Tumor Stage**	Early	26	10 (38.5)	16 (61.5)			
	Late	51	25 (49.0)	26 (51.0)	0.379	0.650	0.248–1.701
**Pattern of Invasion**	Cohesive	18	8 (44.4)	10 (55.6)			
	Non-cohesive	59	27 (45.8)	32 (54.2)	0.922	0.948	0.328–2.741
**Broder's grading**	Well	33	14 (42.4)	19 (57.6)			
	Moderate/poor	40	20 (50.0)	20 (50.0)	0.518	0.737	0.291–1.863

Abbreviations: n, number of specimens.

Data were analyzed by Pearson's Chi-Square test.

*p < 0.05.

Pattern of invasion at the invasive front of tumors has been reported to be associated with outcome where patients whose tumors were found to be non-cohesive had an increased risk for metastasis and poorer survival [9, 39]. In this context, diffused LAMC2 expression was associated with non-cohesive pattern of invasion (*p* = 0.011) and reduced CDH1 expression was trending towards non-cohesive pattern of invasion (*p* = 0.064, Table 3). While EMT has been widely associated with tumor metastasis and invasion in many tumors including OSCC [12, 15, 34, 40–46], we did not observe any significant association between the EMT-related markers examined in this study with nodal metastasis. Furthermore, association between EMT-related markers with tumor stage and tumor size was also not observed.

### Association of EMT-related markers expression with clinical outcome

**Fig 2:**
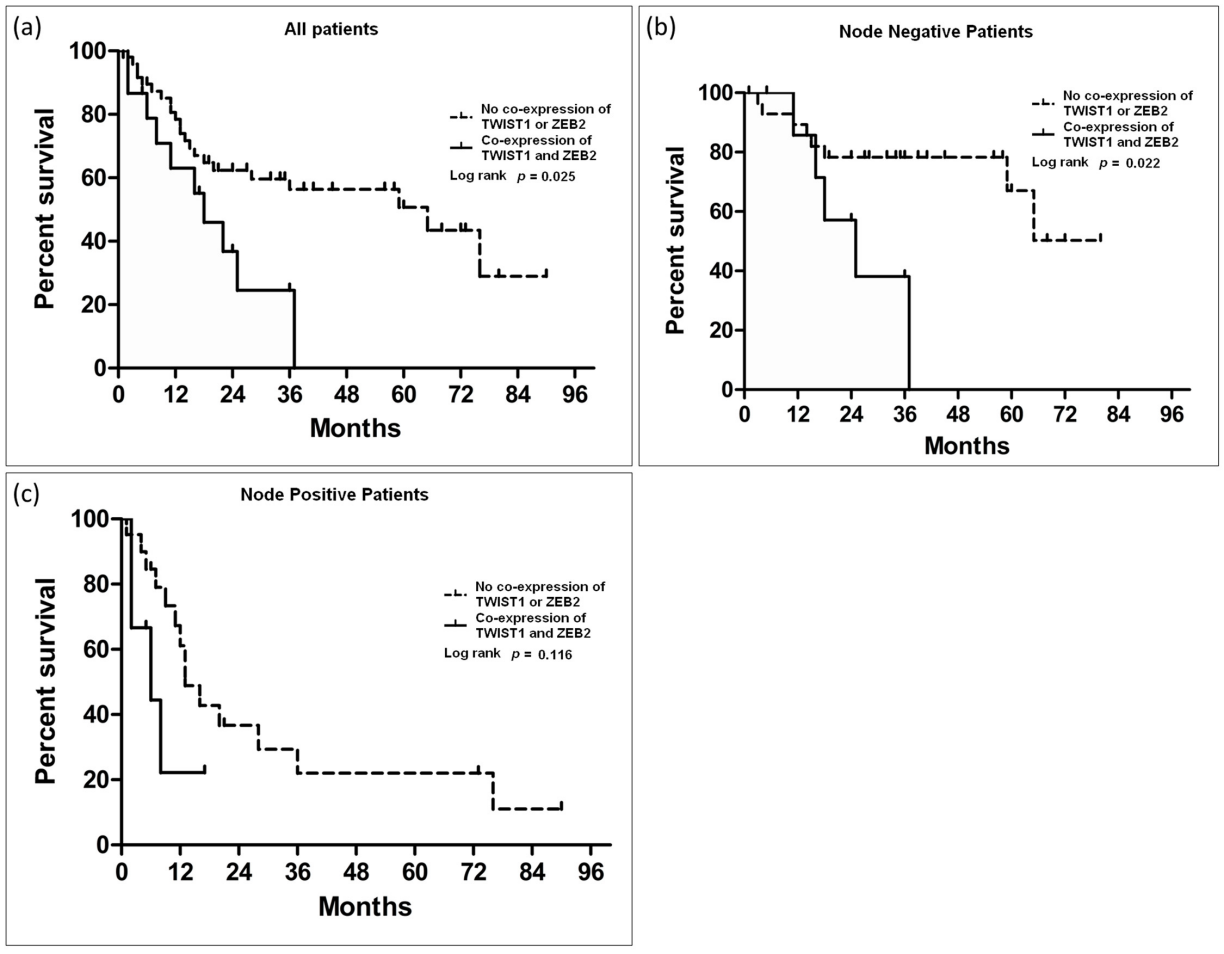
Association of co-expression of TWIST1 and ZEB2 with survival. (a) OSCC patients with co-expression of TWIST1 and ZEB2 have poorer overall survival compared to patients with no co-expression of these two proteins. Co-expression of TWIST1 and ZEB2 is significantly associated with overall survival in node negative OSCC patients (b) but not in node positive OSCC patients (c).

The association between the expressions of EMT-related markers with patient survival was also examined. None of the EMT-related markers, when analyzed alone, were significantly associated with overall survival in OSCC patients (S4 Table). However, as EMT networks are complex and that these proteins could work collectively to drive cancer progression, we examined if co-expression of these proteins could influence patient outcome. Amongst all the EMT-related markers, patients with positive ZEB2 and TWIST1 expression were trending towards poorer overall survival (*p* = 0.079 and *p* = 0.081 respectively; S3A and S3B Fig). With this observation, survival analysis was further carried out to determine whether the co-expression of TWIST1 and ZEB2 was indicative of worse outcome in OSCC patients. Indeed, in combination, the expression of these two markers were significantly associated with poorer overall survival in OSCC patients with a two-year survival probability of 36% as compared to 62% in patients without co-expression of these proteins (*i*.*e*. patients with expression of either marker and patients who were negative for both markers; *p* = 0.025, Fig 2A). Furthermore, multivariate analysis demonstrated that after correction with confounding factors, the co-expression remains an independent prognostic factor and carries a 3.1-fold hazard ratio of poor overall survival (95% CI: 1.314–7.138; *p* = 0.010; Table 4A). As lymph node metastasis is highly associated with poor prognosis [47], we determined the association between the co-expression of TWIST1 and ZEB2 and patient survival in lymph node positive and negative patients separately. The co-expression of TWIST1 and ZEB2 was significantly associated with poor survival in patients without lymph node metastasis with a two-year survival probability of 57.1% as compared to 78.3% in node-negative patients without the co-expression of these proteins (*p* = 0.022, Fig 2B), but this was not observed in patients with positive node metastasis (Fig 2C). Multivariate analysis further demonstrated that the co-expression of TWIST1 and ZEB2 in node-negative patients remains independent prognostic factors with the hazard ratio of 3.8 (95% CI: (1.070–13.247); *p* = 0.039, Table 4B).

**Table 4:** (A) Cox regression analysis demonstrates that co-expression of TWIST1 and ZEB2 is an independent prognostic factor for poor overall survival. (B) Cox regression analysis among patients with negative cervical node demonstrates that co-expression of TWIST1 and ZEB2 is an independent prognostic factor for poor overall survival.

**A**		
**Parameters**	**Hazard Ratio (95% CI)**	***p*-value**
**TWIST1 and ZEB2 (No co-expression vs. Co-expression)**	3.062 (1.314–7.138)	0.010*
**TNM Stage (Early vs. Advance)**	1.445 (0.459–4.544)	0.529
**Lymph node metastasis (Negative vs. Positive)**	2.271 (0.856–6.027)	0.100
**Pattern of invasion (Cohesive vs. Non-Cohesive)**	2.122 (0.632–7.129)	0.224
**B**		
**Parameters**	**Hazard Ratio (95% CI)**	**p-value**
**TWIST1 and ZEB2 (No co-expression vs. Co-expression)**	3.765 (1.070–13.247)	0.039*
**TNM Stage (Early vs. Advance)**	1.198 (0.351–4.085)	0.772
**Pattern of invasion (Cohesive vs. Non-Cohesive)**	2.598 (0.717–9.416)	0.146

Abbreviations: CI, confidence intervals

*p < 0.05.

## Discussion

EMT is an important process in the development of cancer and key features can be indicated by the expression of specific proteins [2, 4]. EMT has been previously reported to occur at the invasive tumor front [7, 12–23] suggesting that this process can likely contribute to the invasive potential of tumor cells. While several studies have investigated the occurrence of EMT in OSCC, these have largely focused only on the loss of CDH1 and the overexpression of LAMC2 [16, 48, 49]. Notably, other important EMT-related markers such as SNAI1, SNAI2, ZEB1, ZEB2 and TWIST1 at the invasive tumor front were not thoroughly examined in these reported studies. In this study, the expression of an extended panel of EMT related markers (*i*.*e*. CDH1, LAMC2, SNAI1/2, ZEB1, ZEB2 and TWIST1) was examined in a large cohort of tumor samples consisting of OSCC at the invasive tumor front of the tumor, in order to shed light on important prognostic information [9–11]. Four of these EMT-related proteins (CDH1, LAMC2, SNAI1/2 and TWIST1) were found to be differentially expressed between OSCC and normal oral mucosal epithelium. Reduced expression of CDH1, a key protein of adherens junctions anchoring oral epithelial cells to one another was observed. This is perhaps not surprising as the loss of CDH1 expression results in the destabilization of adherens junctions and loss of epithelial cell adhesion is an important event in tumorigenesis [50]. CDH1 loss has been frequently reported in OSCC particularly at the invasive tumor front [16, 20, 21], and is well-recognized as one of the hallmarks of EMT.

Another indication that cells may have gained tumorigenic potential is the overexpression of LAMC2. In this study, LAMC2 a basement membrane protein that maintains cell contact integrity by promoting static adhesion and hemidesmosome formation [51], was found to be overexpressed in the cytoplasm of OSCC cells. Notably, 2 distinct patterns of expression (i.e. peripheral and diffused) have been previously reported in OSCC and these pattern of expression is associated with lymph node metastasis and poor prognosis [7, 22, 52]. In this study, the majority of OSCC demonstrated diffused staining at the invasive front of the tumor, consistent with its role in tumor invasion and migration [53].

One of the phenotypes of EMT is the change of cell morphology. This can be controlled by the expression of Snail family of transcription factors which can impact cell shape and affect cell adhesion, resulting in an increased invasive potential [54]. In this study, SNAI1/2 was found to be significantly overexpressed, in greater than 70% of OSCC specimens. Similar to our study, Wushou *et al*. reported that a high number of OSCC showed Snail expression compared with normal epithelium [46]. Notably, Snail controls the expression of matrix metalloproteinases (MMPs) and other transcription factors (*i*.*e*. TWIST1, ZEB1, ZEB2) and therefore could be regarded as a master regulator of the EMT process [50]. In this study, TWIST1 was also found to be overexpressed in OSCC. Consistent with our observation, TWIST1 has been reported to be over-expressed in several cancers including OSCC [45, 55]. In this study, 26% OSCC showed overexpression of TWIST1 suggesting that TWIST1 could be an important protein in a subset of OSCC patients, as also demonstrated by Sakamoto and colleagues [45].

The current understanding of the EMT process is that transcription factors including SNAI1, SNAI2, ZEB1, ZEB2 and TWIST1 down-regulate CDH1 expression which subsequently results in the loss of cell adhesion and migration of tumor cells [4]. However, emerging evidence suggests that transcription repression of CDH1 may not be a universal phenomenon as an inverse association between the expression of CDH1 and EMT transcription factors is not always observed [40, 45, 56]. Consistent with this, our study did not find any association between the expression of CDH1 and the transcription factors despite the fact that CDH1 was observed to be down-regulated in the majority (64.9%) of OSCC specimens. In addition to being repressed by EMT-related proteins, CDH1 expression can be modulated by several other mechanism including genomic deletion, loss of heterozygosity and promoter hypermethylation [45, 57, 58], possibly explaining mechanisms of CDH1 down-regulation observed in this study.

Next, we examined the correlation between the expressions of these proteins with clinico-pathological parameters, which could provide clues on the role of each protein, and could identify useful biomarkers for prognostication. In this study, we found that diffused LAMC2 was associated with non-cohesive pattern of invasion while CDH1 loss was trending towards non-cohesive pattern of invasion at the OSCC invasive front. This observation is consistent with previous studies that showed that LAMC2 overexpression and CDH1 loss were also observed in infiltrative strands or small nests of tumor cells and tumor buds in oral and pancreatic cancers [7, 16, 18, 23, 59] suggesting that LAMC2 and CDH1 proteins could participate in a phenomenon known as tumor budding which is important for the ability of tumor cells to migrate and invade [16, 48]. In addition, reduced CDH1 was also significantly observed in less differentiated OSCC. This is perhaps not unexpected as CDH1 has an important role in maintaining the structural and functional integrity of epithelium and upon the loss of CDH1, epithelial cell polarity and cell adhesion are lost [30], giving rise to a phenotype that is frequently observed in cells that are invading [30]. Interestingly, we did not see an association between the expression of several EMT-related proteins and lymph node metastasis. While SNAI1/2 overexpression in different tumors (head and neck, lung, pancreas, breast) were significantly associated with node metastasis [15, 34, 40, 42, 44, 60], other studies showed no association [46, 61, 62]. Similarly, the association between CDH1 loss and node metastasis is still inconclusive, whether these contradictory findings are associated with etiological factors remains to be examined [12, 20, 23, 63].

The occurrence of EMT has been shown to indicate an aggressive disease and therefore, we hypothesized that expression patterns of EMT-related proteins could be associated with worse prognosis in our OSCC patient cohort. Indeed, we observed a trend where TWIST1 and ZEB2 were associated with poor prognosis, although this did not reach statistical significance. As the EMT process is complex and often involves more than one EMT-related protein [2, 4, 50], it is likely that simultaneous overexpression of several EMT-related proteins are necessary for tumorigenesis. Taking into the account the co-expression of TWIST1 and ZEB2, we found that patients with expression of both proteins had significantly poorer survival compared to those whose tumor only expressed one or none of the 2 proteins. This effect was more apparent when we examined the expression of these 2 proteins amongst lymph node negative patients where individuals with co-expression of TWIST1 and ZEB2 are 3.8 times more likely to have a poor prognosis compared to those with the expression of either one or neither of the proteins indicating that the co-expression of these 2 markers is an independent prognostic factor for identifying aggressive disease particularly amongst lymph node negative patients. Emerging evidence suggest that EMT-related markers have other oncogenic roles in addition to driving the EMT process as measured by change of cell morphology and invasion. Although studying the functional role of EMT proteins is not within the scope of this study, previous studies demonstrated that TWIST1 and ZEB2 have other roles including repressing cellular senescence and conferring stem-like properties to cancer cells respectively [64, 65].

Currently, histopathological assessment of surgical specimens according to the American Joint Committee on Cancer and Union for International Cancer Control (AJCC and UICC) staging criteria remains the gold standard in determining the extent of the disease, and continues to provide crucial information in determining the prognosis of the patient for treatment planning. However, it is well accepted that patients with the same disease stage do not response uniformly to similar treatment strategies and this is in part due to the inherent genetic drivers that are unique to each tumor [66, 67]. While lymph node metastasis remains the most relevant clinical prognosticator, the need to identify appropriate management for high risk individuals with no detectable lymph node metastasis at the point of diagnosis is important [52]. Therefore, the co-expression of TWIST1 and ZEB2 could be particularly important in identifying patients without detectable regional metastasis but nevertheless should be treated aggressively. Notably, both expressions of TWIST1 and ZEB2 have been reported to be of significant prognostic value in early stage colorectal and oral cancer patients where disease spread is not always evident [68, 69]. The mechanism by which this segregation happens is still unclear and further investigation is needed, but perhaps, co-expression of TWIST1 and ZEB2 could identify patients with undetected tumor spread. These observations will require further validation in an independent cohort of patient specimens to ensure its robust use in the clinical setting.

In summary, following closely to the REMARK criteria [70], we report that EMT-related markers are frequently differentially expressed between OSCC and NOM tissues. Consistent with their known function, expression of some of these proteins were associated with clinico-pathological phenotypes. Notably, co-expression of TWIST1 and ZEB2 was significantly prevalent in OSCC patients with poorer overall survival particularly in patients with no lymph node metastasis. Further prospective studies focusing on these two transcription factors should be able to determine their clinical value in identifying patients with poor survival for appropriate management.

## Supporting Information

S1 Fig Representative immune-staining of EMT-related proteins in different cancers as positive controls. SNAI1/2 in breast cancer (a), ZEB2 in colorectal cancer (b), ZEB1 in colorectal cancer (c), TWIST1 in cervical cancer (d), CDH1 in breast cancer (e), LAMC2 in breast cancer (f), ZEB1 in breast cancer (g) and ZEB1 in ovarian cancer (h) (Original magnification: 400X).
(TIF)

S2 Fig Representative Immunostaining of EMT-related proteins in TMA cores and respective whole tissue section for concordance evaluation (Original magnification: 400X). (TIF)

S3 Fig Kaplan Meier survival analyses of ZEB2 and TWIST1. Near significant association of expression of ZEB2 (a) and TWIST1 (b) with poor patient’s overall survival.
(TIF)

S1 Table Immunohistochemical staining protocol and evaluation for antibody against EMT-related proteins. (XLSX)

S2 Table Sample size calculation. (XLSX)

S3 Table Correlation between CDH1 expression with expression of EMT transcription factors. (XLSX)

S4 Table Association between expression of EMT-related proteins with overall survival. (XLSX)
