# Supplementary figures and images for: Co-Expression of TWIST1 and ZEB2 in Oral Squamous Cell Carcinoma Is Associated with Poor Survival
Source: PLoS One. 2015 Jul 27;10(7):e0134045. doi: 10.1371/journal.pone.0134045 (PMC4516250; doi:10.1371/journal.pone.0134045)

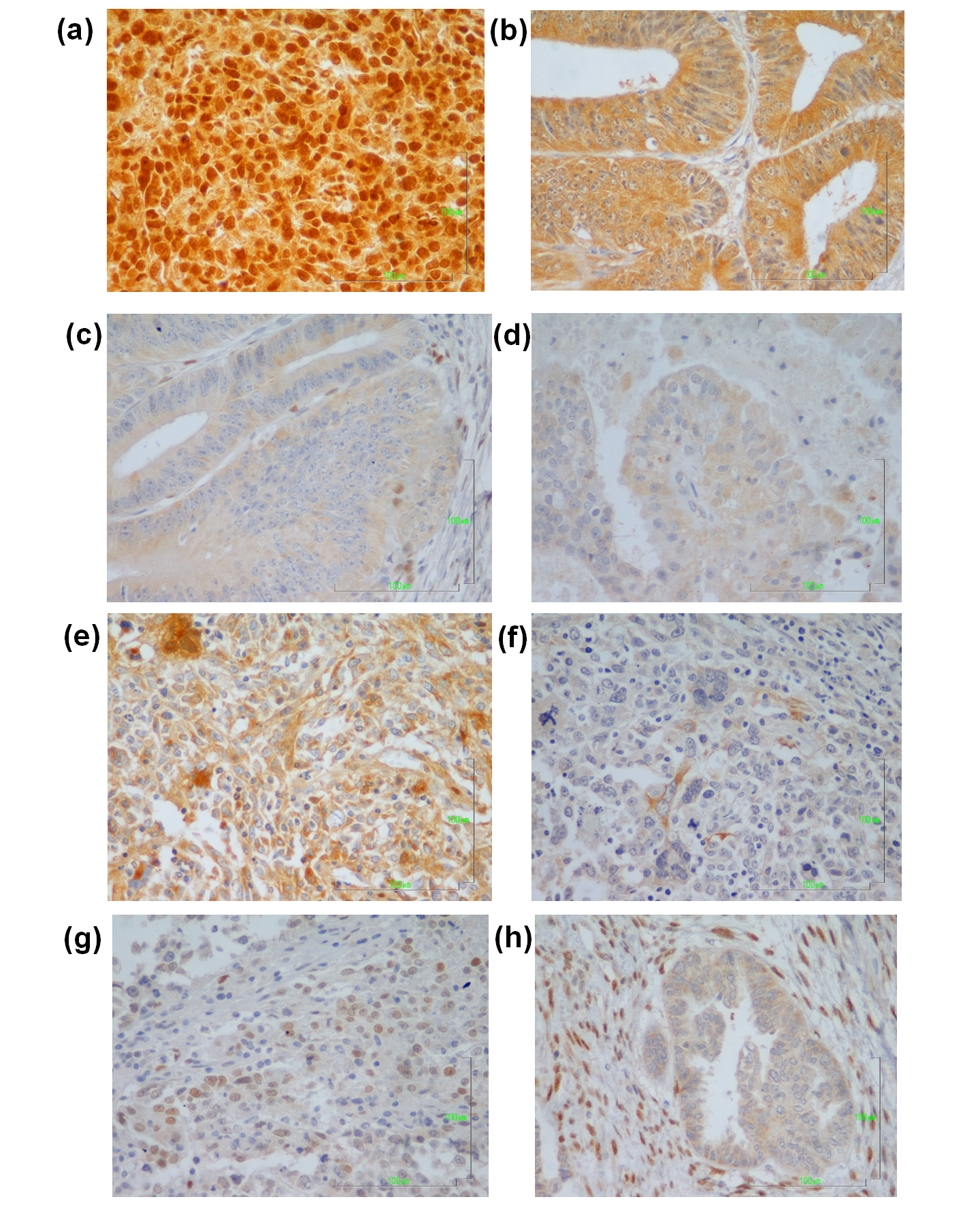

Supplement: S1 Fig — SNAI1/2 in breast cancer (a), ZEB2 in colorectal cancer (b), ZEB1 in colorectal cancer (c), TWIST1 in cervical cancer (d), CDH1 in breast cancer (e), LAMC2 in breast cancer (f), ZEB1 in breast cancer (g) and ZEB1 in ovarian cancer (h) (Original magnification: 400X). (TIF) [file pone.0134045.s001.tif]

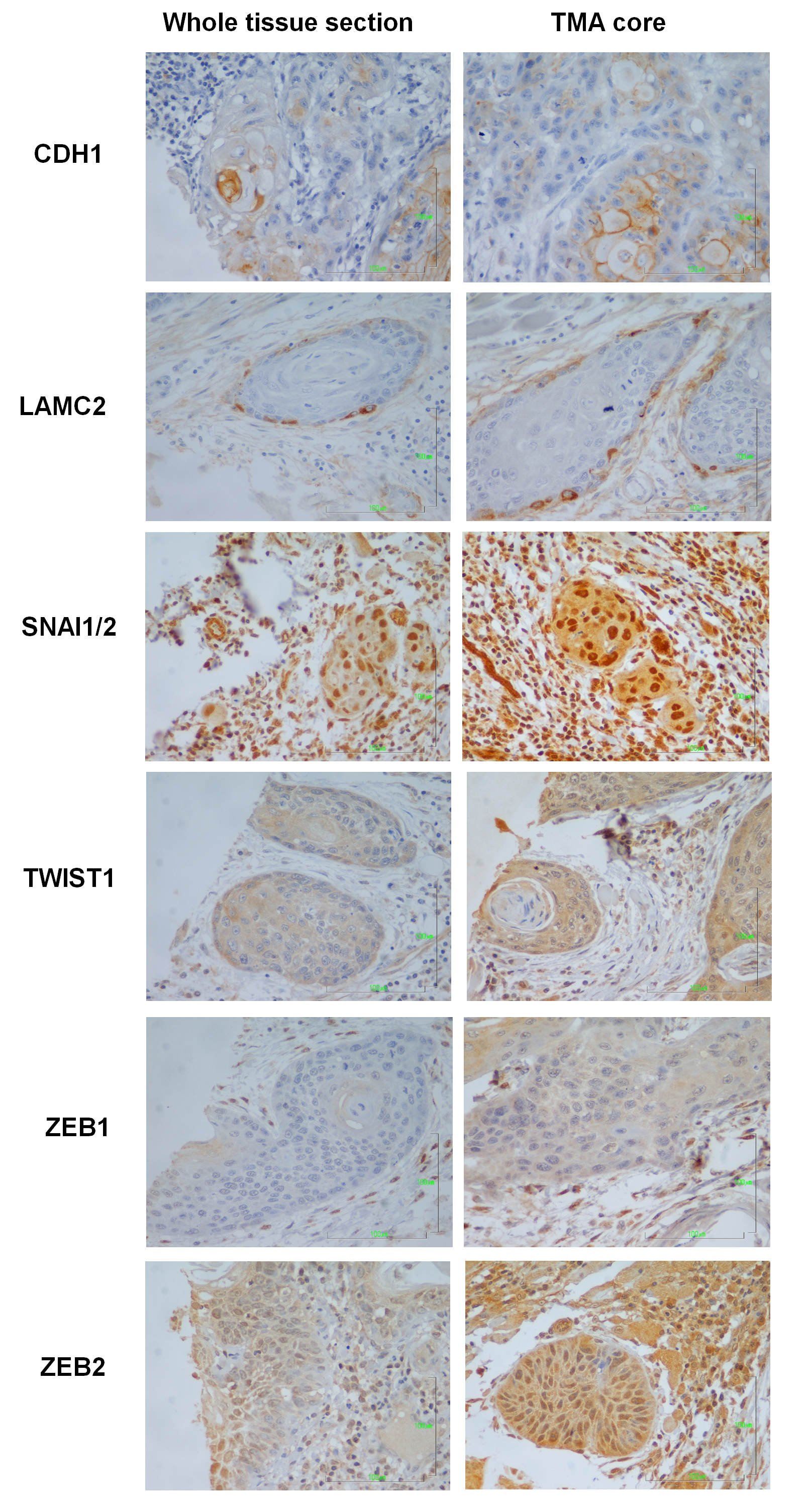

Supplement: S2 Fig — (TIF) [file pone.0134045.s002.tif]

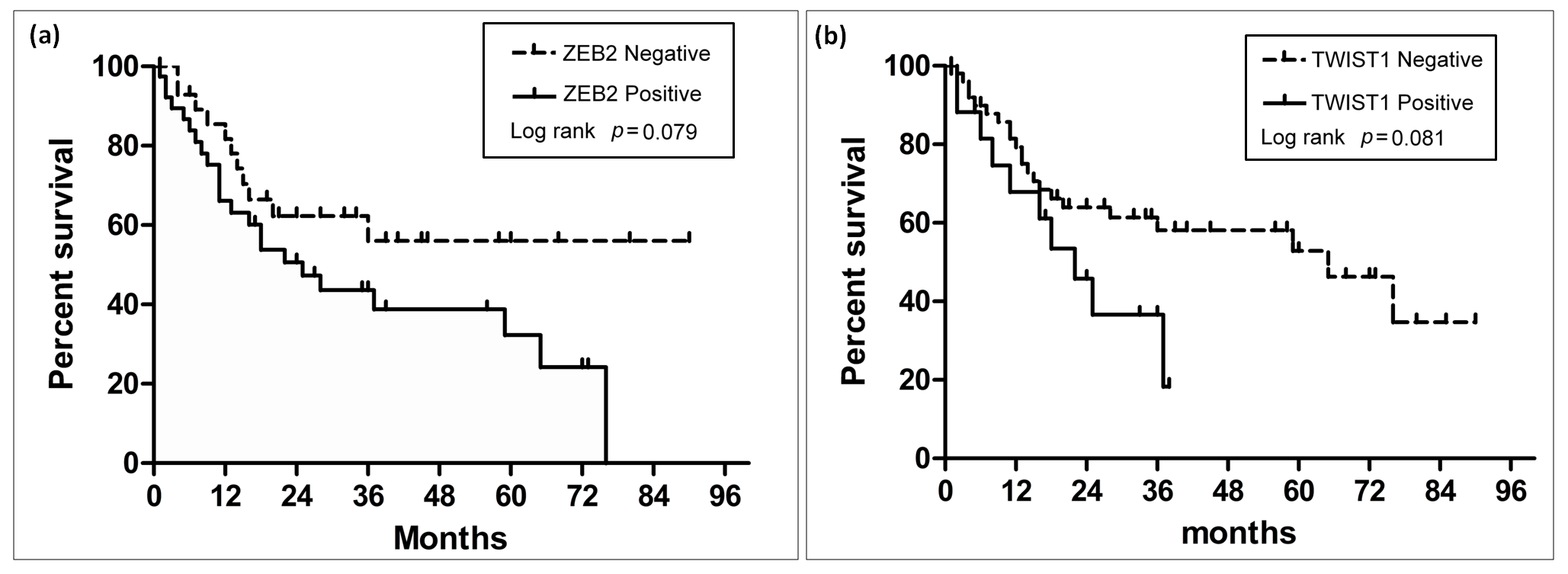

Supplement: S3 Fig — Near significant association of expression of ZEB2 (a) and TWIST1 (b) with poor patient’s overall survival. (TIF) [file pone.0134045.s003.tif]
